# Assessment of Radiation Dose in Medical Imaging and Interventional Radiology Procedures for Patient and Staff Safety

**DOI:** 10.3390/diagnostics11061116

**Published:** 2021-06-18

**Authors:** Kosuke Matsubara

**Affiliations:** Department of Quantum Medical Technology, Faculty of Health Sciences, Institute of Medical, Pharmaceutical and Health Sciences, Kanazawa University, 5-11-80 Kodatsuno, Kanazawa, Ishikawa 9200942, Japan; matsuk@mhs.mp.kanazawa-u.ac.jp; Tel.: +81-76-265-2500

Medical imaging and interventional radiology procedures that use ionizing radiation play a significant role in patient healthcare. However, the biologic effects of X-ray exposures related to medical imaging and interventional radiology procedures have been investigated and debated for a long time.

International Commission on Radiological Protection (ICRP) suggested three fundamental principles of radiation protection: justification, optimization of protection, and application of dose limits [1]. With regard to medical radiation exposure to patients, the dose limits are not applied because this type of exposure is intentional and for the direct benefit of the patient. Instead, the emphasis is then on justification of the medical procedures and on optimization of radiation protection in order to minimize radiation risk to patients. The principle of as low as reasonably achievable (ALARA) has long provided a basic guideline for medical imaging. Especially, radiation doses in computed tomography (CT) are nontrivial and the number of individuals being exposed is large and rapidly increasing. With regard to occupational radiation exposure, which is defined as all radiation exposure of workers incurred as a result of their work, the application of dose limits is important in order to prevent deterministic effects and limit the occurrence of stochastic effects to a tolerable level.

During the process of the optimization of protection in medical imaging, the use of modern imaging systems and efficient reconstruction techniques is shown to be effective in order to reduce patient radiation dose. A deep learning reconstruction significantly reduced image noise and improved overall image quality of CT pulmonary angiography examinations in the emergency setting and offered an additional significant radiation dose reduction while allowing slices to be twice as thin as compared to hybrid iterative reconstruction (IR) [2]. The use of an Advanced Modeled IR (ADMIRE; Siemens Healthineers, Forchheim, Germany) allowed a reduction in the radiation dose to 25% of the original dose with the same diagnostic accuracy for the assessment of neck abscesses [3]. Paired inspiratory-expiratory scans were able to be acquired on 3rd generation dual source CT (DSCT) system (Somatom Force; Siemens Healthineers, Erlangen, Germany) at substantially lower dose and risk levels when compared to inspiration-only scans at conventional CT systems, offering promising prospects for improved chronic obstructive pulmonary disease diagnosis [4]. The 3rd generation DSCT system also maintained good subjective image quality, diagnostic confidence, and image noise in single-energy whole-body staging at dose levels as low as 40% of the original dose, when using ADMIRE (with strength 3) [5].

Diagnostic reference levels (DRLs) have been proven to be an effective tool that aids in the optimization of protection in the medical radiation exposure of patients for medical imaging and interventional radiology procedures [6]. Radiation exposure and image noise varied widely among children of different ages, and there might be a need to establish DRLs for specific age groups [7]. The local DRLs of a local healthcare institution were established with the inclusion of the noise magnitude [8]. The local DRLs for adult patients undergoing head, chest, and abdominal scans were established and compared with the published national DRLs, and the impact of factors influencing CT dose metrics was investigated through multivariate analysis in order to facilitate the modification of CT imaging procedures once the local DRLs are unusually high compared to the national DRLs [9].

The ICRP expressed concern about the use of CT in advanced medicine and the fact that it can cause a cancer risk relative to other imaging methods [1]. The effective risk associated with CT pulmonary angiography examinations was estimated regarding body diameter and sex, and the results showed that the effective risk for male subjects was slightly lower than for female subjects [10]. It is important to balance the requirement for contrast with a reduction in the patient dose during full-spinal radiographs, although a high tube voltage and the removal of the anti-scatter grid were effective for reducing the breast dose [11]. Effective dose conversion factors that were based on CT examination data obtained from a nationwide survey in the Republic of Korea were determined, and they were expected to be allowed for a more accurate benefit-risk assessment of CT imaging [12].

The most important factor for managing patient dose during interventional radiology procedures is to evaluate the maximum skin dose in order to prevent skin injuries. Radiation doses associated with endovascular treatments such as thoracic endovascular aortic repair and endovascular aortic repair using a hybrid operating room system, which is increasingly being used to perform endovascular procedures, were determined, and the results showed that some patients were exposed to >2 Gy as the air kerma at the patient entrance reference point (K_a,r_) [13]. During cerebral angiography including neurointerventional radiology procedures, it was suggested that the quadratic function for the K_a,r_ ratio provided a more accurate estimate of the maximum skin dose in real time [14].

A new threshold dose of 0.5 Gy was suggested for radiation effects on the eye lens and a new occupational equivalent dose limit for the eye lens of 20 mSv/year, averaged over defined periods of five years, with no single year exceeding 50 mSv, was recommended by ICRP [15]. Small-type radiophotoluminescence glass dosimeters and optically stimulated luminescence dosimeters had different dosimeter properties, and they influenced measured eye lens doses for the physician, especially on the opposite side of the patient [16]. Physician and staff doses to eye lenses associated with CT-guided interventional procedures were not significant when they were performed with appropriate radiation protection and low-dose techniques [17].

For a patient undergoing a medical imaging or an interventional radiology procedure, both the amount of radiation that is incident on the patient and the total radiation received by the patient need to be determined. This information can be used for the derivation of local and total dose quantities that are related to deterministic and stochastic risks to the patient. Not only patient dose, but also radiation doses for medical staff, need to be evaluated with appropriate methods for improving the safety of medical staff. I believe this special issue has provided several important points in order to minimize radiation risks to patients and medical staff.

## References

[1] ICRP (2007). The 2007 Recommendations of the International Commission on Radiological Protection. ICRP Publication 103. Ann ICRP.

[2] Lenfant M., Chevallier O., Comby P.-O., Secco G., Haioun K., Ricolfi F., Lemogne B., Loffroy R. (2020). Deep Learning Versus Iterative Reconstruction for CT Pulmonary Angiography in the Emergency Setting: Improved Image Quality and Reduced Radiation Dose. Diagnostics.

[3] Winkelmann M.T., Afat S., Walter S.S., Stock E., Schwarze V., Brendlin A., Kolb M., Artzner C.P., Othman A.E. (2020). Diagnostic Performance of Different Simulated Low-Dose Levels in Patients with Suspected Cervical Abscess Using a Third-Generation Dual-Source CT Scanner. Diagnostics.

[4] Gawlitza J., Henzler T., Trinkmann F., Nekolla E., Haubenreisser H., Brix G. (2020). COPD Imaging on a 3rd Generation Dual-Source CT: Acquisition of Paired Inspiratory-Expiratory Chest Scans at an Overall Reduced Radiation Risk. Diagnostics.

[5] Brendlin A.S., Winkelmann M.T., Do P.L., Schwarze V., Peisen F., Almansour H., Bongers M.N., Artzner C.P., Weiss J., Kim J.H. (2021). Simulated Radiation Dose Reduction in Whole-Body CT on a 3rd Generation Dual-Source Scanner: An Intraindividual Comparison. Diagnostics.

[6] ICRP (2017). Diagnostic Reference Levels in Medical Imaging. ICRP Publication 135. Ann ICRP.

[7] Muhammad N.A., Abdul Karim M.K., Abu Hassan H., Ahmad Kamarudin M., Ding Wong J.H., Ng K.H. (2020). Diagnostic Reference Level of Radiation Dose and Image Quality among Paediatric CT Examinations in A Tertiary Hospital in Malaysia. Diagnostics.

[8] Harun H.H., Abdul Karim M.K., Abd Rahman M.A., Abdul Razak H.R., Che Isa I.N., Harun F. (2020). Establishment of CTPA Local Diagnostic Reference Levels with Noise Magnitude as a Quality Indicator in a Tertiary Care Hospital. Diagnostics.

[9] Yang C.-C. (2020). Evaluation of Impact of Factors Affecting CT Radiation Dose for Optimizing Patient Dose Levels. Diagnostics.

[10] Harun H.H., Abdul Karim M.K., Abbas Z., Abdul Rahman M.A., Sabarudin A., Ng K.H. (2020). Association of Radiation Doses and Cancer Risks from CT Pulmonary Angiography Examinations in Relation to Body Diameter. Diagnostics.

[11] Nemoto M., Chida K. (2020). Reducing the Breast Cancer Risk and Radiation Dose of Radiography for Scoliosis in Children: A Phantom Study. Diagnostics.

[12] Lee S.-K., Kim J.S., Yoon S.-W., Kim J.M. (2020). Development of CT Effective Dose Conversion Factors from Clinical CT Examinations in the Republic of Korea. Diagnostics.

[13] Haga Y., Chida K., Sota M., Kaga Y., Abe M., Inaba Y., Suzuki M., Meguro T., Zuguchi M. (2020). Hybrid Operating Room System for the Treatment of Thoracic and Abdominal Aortic Aneurysms: Evaluation of the Radiation Dose Received by Patients. Diagnostics.

[14] Morota K., Moritake T., Nagamoto K., Matsuzaki S., Nakagami K., Sun L., Kunugita N. (2021). Optimization of the Maximum Skin Dose Measurement Technique Using Digital Imaging and Communication in Medicine—Radiation Dose Structured Report Data for Patients Undergoing Cerebral Angiography. Diagnostics.

[15] ICRP (2012). ICRP Statement on Tissue Reactions / Early and Late Effects of Radiation in Normal Tissues and Organs—Threshold Doses for Tissue Reactions in a Radiation Protection Context. Ann ICRP.

[16] Matsubara K., Yoshida S., Hirosawa A., Chusin T., Furukawa Y. (2021). Characterization of Small Dosimeters Used for Measurement of Eye Lens Dose for Medical Staff during Fluoroscopic Examination. Diagnostics.

[17] Inaba Y., Hitachi S., Watanuki M., Chida K. (2021). Occupational Radiation Dose to Eye Lenses in CT-Guided Interventions Using MDCT-Fluoroscopy. Diagnostics.

